# Gene expression changes during caste-specific neuronal development in the damp-wood termite *Hodotermopsis sjostedti*

**DOI:** 10.1186/1471-2164-11-314

**Published:** 2010-05-20

**Authors:** Yuki Ishikawa, Yasukazu Okada, Asano Ishikawa, Hitoshi Miyakawa, Shigeyuki Koshikawa, Toru Miura

**Affiliations:** 1 Laboratory of Ecological Genetics, Graduate School of Environmental Science, Hokkaido University, Sapporo, Hokkaido, 060-0810, Japan; 2 Laboratory of Evolutionary Ecology, Graduate School of Environmental Science, Okayama University, 700-8530, Tsushima-naka 1-1-1, Okayama, Japan; 3 Laboratory of Molecular Biology, University of Wisconsin-Madison, 1525 Linden Drive, Madison, Wisconsin 53706, USA

## Abstract

**Background:**

One of the key characters of social insects is the division of labor, in which different tasks are allocated to various castes. In termites, one of the representative groups of social insects, morphological differences as well as behavioral differences can be recognized among castes. However, very little is known about the neuronal and molecular bases of caste differentiation and caste-specific behavior. In almost all termite species, soldiers play defensive roles in their colonies, and their morphology and behavior are largely different from workers (or pseudergates). Therefore, we predicted that some genes linked to defensive behavior and/or those required for neuronal changes are differentially expressed between workers and soldiers, or during the soldier differentiation, respectively.

**Results:**

Using the brain and suboesophageal ganglion (SOG) of the damp-wood termite *Hodotermopsis sjostedti*, we first screened genes specifically expressed in soldiers or during soldier differentiation by the differential display method, followed by quantitative real-time polymerase chain reaction. No distinctive differences in expression patterns were detected between pseudergates and soldiers. In the course of soldier differentiation, however, five genes were found to be up-regulated in brain and/or SOG: 14-3-3epsilon, fibrillin2, beta-tubulin, ciboulot, and a hypothetical protein containing a SAP motif. Some of these genes are thought to be associated with cytoskeletal structure or motor-associated proteins in neuronal tissues.

**Conclusion:**

The identified five genes could be involved in soldier-specific neuronal modifications, resulting in defensive behaviors in termite soldiers. The temporal expression patterns of these genes were consistent with the neuronal changes during soldier differentiation, suggesting that molecular machineries, in which the identified factors would participate, play important roles in behavioral differentiation of termite soldiers.

## Background

Social insects exhibit well-organized social behavior. Colony members show caste-specific behavioral variations and are specialized in certain tasks [1]. The behavioral differentiation among castes is one of the most important mechanisms to integrate social behaviors, together with social communications. Recent approaches to understand the molecular bases underlying social behaviors have revealed that differential gene expressions contribute to caste differentiation in some social insects [2-5]. However, most of those recent studies were concentrated on the honeybee and other social hymenopterans, while only a few studies have been pursued in other social lineages. To understand the general principles of sociality in insects, extensive studies on the molecular mechanisms in various social insects would be required.

Termites are the oldest eusocial insects and have developed elaborate social systems independently of hymenopterans [6]. The social structures of termites are different from those of social hymenopterans in many aspects [7,8]. One of the most fundamental reasons for the differences is that termites are hemimetabolous, whereas social hymenopterans are holometabolous. Young termites possess similar body patterns to imagos and thus move around just after they are hatched; therefore, relatively younger instar juveniles can function as workers. In contrast, juveniles of social hymenopterans require care of workers throughout their larval and pupal stages, and all castes, including sterile and fertile ones, are "adult" individuals [9]. These developmental differences with or without metamorphosis trigger the differences in caste systems at least to some extent [7,8]. In the case of termite development, sterile castes are thought to comprise immature individuals, so that some castes can differentiate into others through molting events [10,11]. One of the most representative examples is soldier differentiation, in which extensive morphological modification occurs, resulting in soldier-specific weapons such as exaggerated mandibles and/or frontal glands [12,13]. With regard to evolution, the soldier caste is thought to have first occurred in the ancestral termite and remain highly conserved across all present-day species [14]. In other words, the acquisition of soldier caste is one of the key evolutionary changes in termite lineages.

In accordance with morphological modifications, termite soldiers also change their behavioral characteristics dramatically. Workers perform helper tasks like foraging, nursing, and nest maintenance whereas soldiers show extensive defensive behavior [13,15]. In response to predatory invasion, workers tend to escape rapidly into the nest and/or show building behavior, whereas soldiers tend to aggregate outside the nest and aggressively attack enemies [15]. This behavioral differentiation is accomplished by molting events from worker (or larva) to soldier via the presoldier stage, and so can be regarded as age polyethism [16]. In contrast, although honeybee workers also change their tasks from nursing to defensive or foraging behaviors depending on their age, that change is more plastic and is unaccompanied by morphological modifications [17,18].

Juvenile hormone (JH) is known to play important roles in soldier differentiation [19,20]. From classic studies to recent molecular studies on termite caste differentiation, many researchers have focused on soldier differentiation because it can be artificially induced by applying juvenile hormone and its analogues [21,22]. This induction method is useful for analyzing the developmental bases of soldier differentiation because individuals undergoing the differentiation are rare under natural conditions. Additionally, extensive histological studies have been carried out using this system [23-25]. Furthermore, by applying recent molecular techniques to the induction experiments, some genes that are thought to be responsible for the physiological and morphological changes have been shown to be up-regulated during soldier differentiation [26-32]. However, nothing has been elucidated yet about the molecular underpinnings of soldier-specific behavior and neuronal modifications, although the behavioral difference between soldiers and workers is remarkable.

To understand the molecular basis underlying the behavioral and neuronal differences between soldiers and workers in termites, we screened the genes in nervous systems, which were differentially expressed between the two castes, or up-regulated during the course of soldier differentiation in damp-wood termite *H. sjostedti*. Using differential display followed by quantitative real-time polymerase chain reaction (qRT-PCR), several genes were identified as candidate genes potentially responsible for soldier-specific defensive behavior.

## Results and Discussion

### Gene screening by differential display

Because the availability of genomic information for *H. sjostedti *was limited, we chose fluorescent differential display (FDD) followed by qRT-PCR to identify differential gene expressions in nervous system, which is responsible for caste-specific behaviors in soldiers.

**Figure 1:**
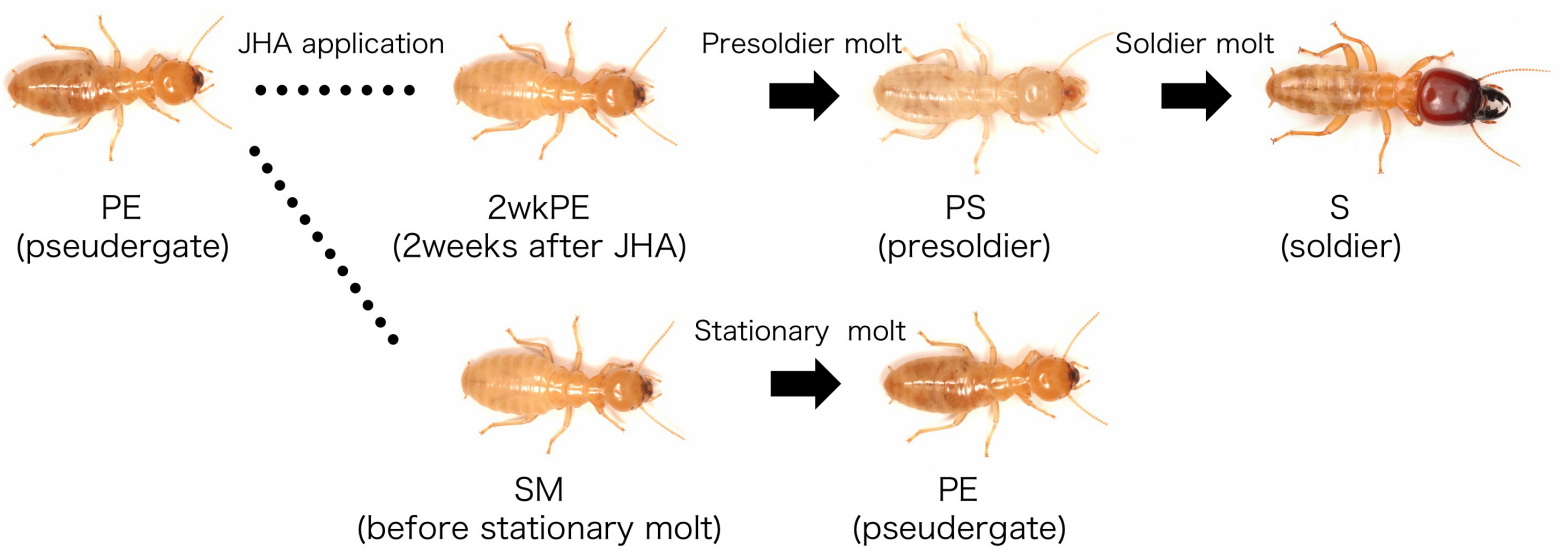
**Soldier differentiation and stationary molt of *H. sjostedti***. Soldier differentiation of the focal termite species can be artificially induced by JHA (pyriproxyfen) application. In this species, 6-7th instar larvae function as workers, i.e., pseudergates (PE). At 2 weeks after the JHA application, abdomens of pseudergates before the presoldier molt turn white because of gut purge (2wkPE). In 2 to 3 weeks after the application, the JHA-applied pseudergates molt into presoldiers (PS). Almost all pseudergates without artificial induction underwent stationary molt (SM).

For the first screening to identify candidate transcripts potentially related to behavioral differences between workers and soldiers, we used mRNA from the brain and SOG (suboesophageal ganglion) of pseudergates (PE; which function as workers; see Methods), soldiers (S), and individuals during soldier differentiation that were artificially induced by applying JHA (JH analogue, pyriproxyfen) [22]. During the differentiation, total RNA was extracted from the brains and SOGs of 2wkPE (pseudergate, 2 weeks after JHA application) and PS (presoldier, 3 weeks after JHA application) because many histological events occur around 2 weeks after the application, and most of the JHA-applied pseudergates molt into presoldiers at around 3 weeks (Figure 1) [25].

**Figure 2:**
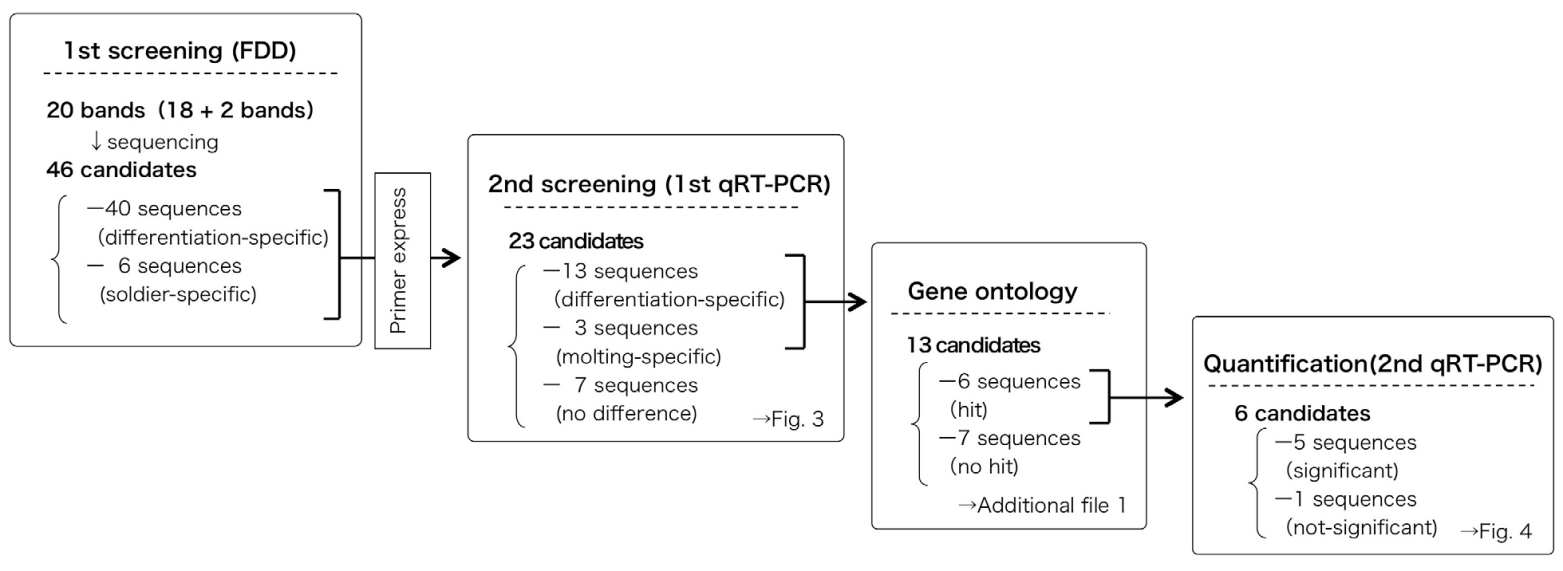
**Screening of soldier- or differentiation-specific genes**. We performed differential display for the first screening and obtained 40 differentiation-specific and 6 soldier-specific gene candidates. Among these, the expressions of 23 candidates were screened by qRT-PCR (second screening, first qRT-PCR). Thirteen of these were confirmed to be up-regulated during soldier differentiation. Based on Gene Ontology and the BLAST-X algorithm, molecular function terms and biological process terms were assigned to six sequences, which were subsequently quantified by qRT-PCR ("Quantification, 2nd qRT-PCR" in Figure 2).

FDD identified 18 candidate bands that specifically appeared during the soldier differentiation (differentiation-specific) and two bands that appeared after the completion of differentiation (soldier-specific). In the process of cloning, four or five clones were sequenced for each candidate band and multiple sequences were occasionally identified from a single band, resulting in 46 different sequences: 40 differentiation-specific and 6 soldier-specific sequences (Figure 2).

### Gene expression profiles examined by qRT-PCR

**Figure 3:**
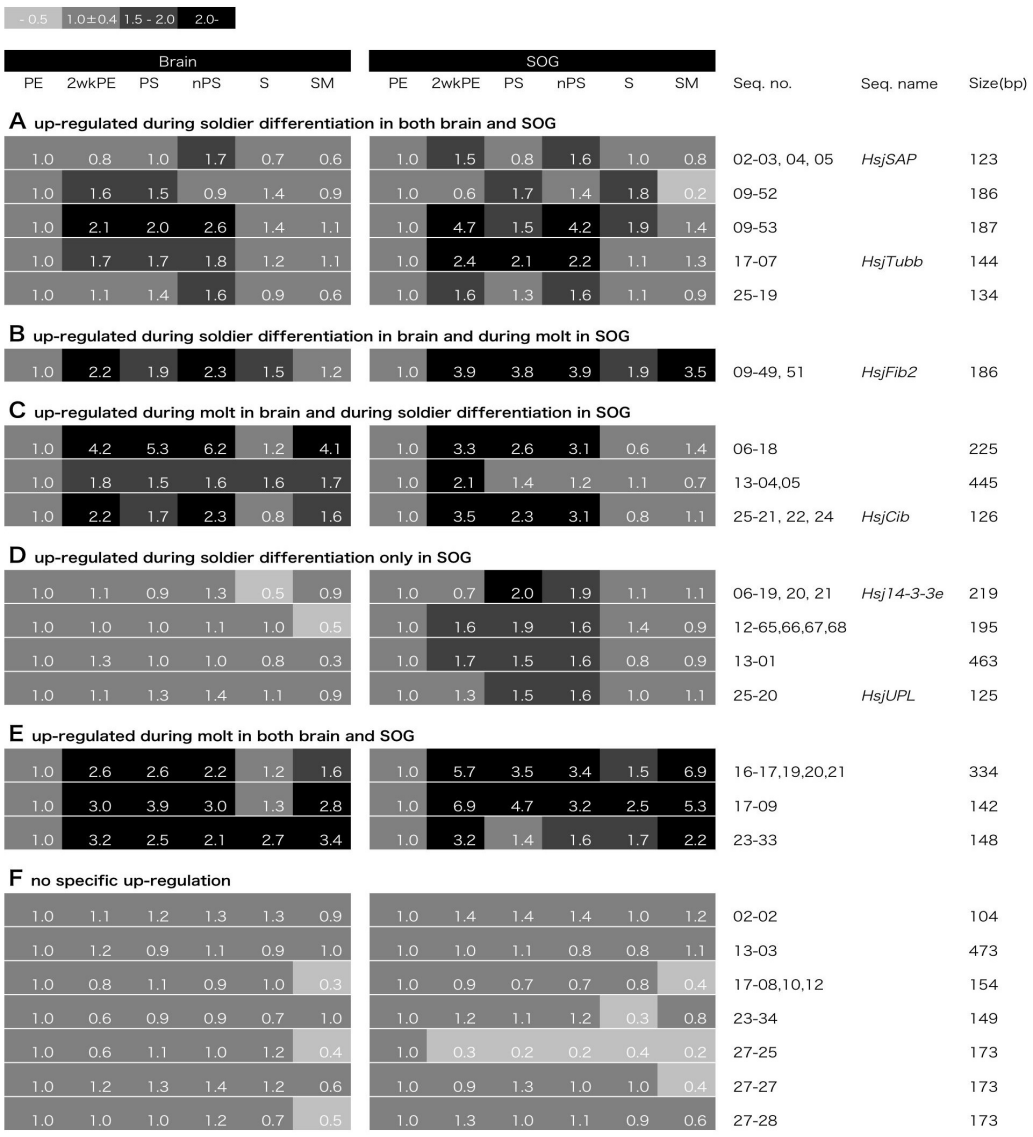
**Gene expression patterns of candidate genes quantified by qRT-PCR**. As a result of the first qRT-PCR, sequences of 23 candidate genes were classified by gene expression patterns. Up-regulated gene candidates were categorized into six groups based on the expression patterns. A: up-regulated during soldier differentiation (2wkPE, PS, or nPS) in both of brain and SOG; B: up-regulated during soldier differentiation in brain and during molt (2wkPE, PS, or nPS, and also SM) in SOG; C: up-regulated during molt in brain and during soldier differentiation in SOG; D: up-regulated during soldier differentiation only in SOG; E: up-regulated during molt in both brain and SOG; F: no specific up-regulation, i.e., false positive.

Next, we performed the second screening using qRT-PCR (first round of qRT-PCR) (Figure 3). In addition to the sample categories (castes/stages) used in FDD, we included "pseudergates during stationary molt (SM)" as the control molt and "natural presoldiers (nPS)" for the evaluation of artificial effects of JHA. qRT-PCR evaluations were performed only on 23 candidates, for which it was possible to design the qRT-PCR primers (Figure 2). Among them, 13 candidates (13/23) were up-regulated more than 1.5-fold during the soldier differentiation, i.e., in 2wkPE, PS, and/or nPS (Figure 3A-D). Among the 13 candidates, five were up-regulated both in the brain and SOG (Figure 3A), whereas only one was up-regulated in the brain (Figure 3B) and the remaining seven candidates in the SOG (Figure 3C,D). Three other candidates (3/23) were also identified as up-regulated genes but also highly expressed in SM, suggesting that they would be generally required for molting irrespective of caste phenotype or developmental stage (Figure 3E). No soldier-specific candidate showing higher expression after the differentiation was identified. Seven candidate genes (7/23) showed similar expression levels (less than 1.5-fold) during the soldier differentiation, so they were excluded from further analyses (Figure 3F).

**Figure 4:**
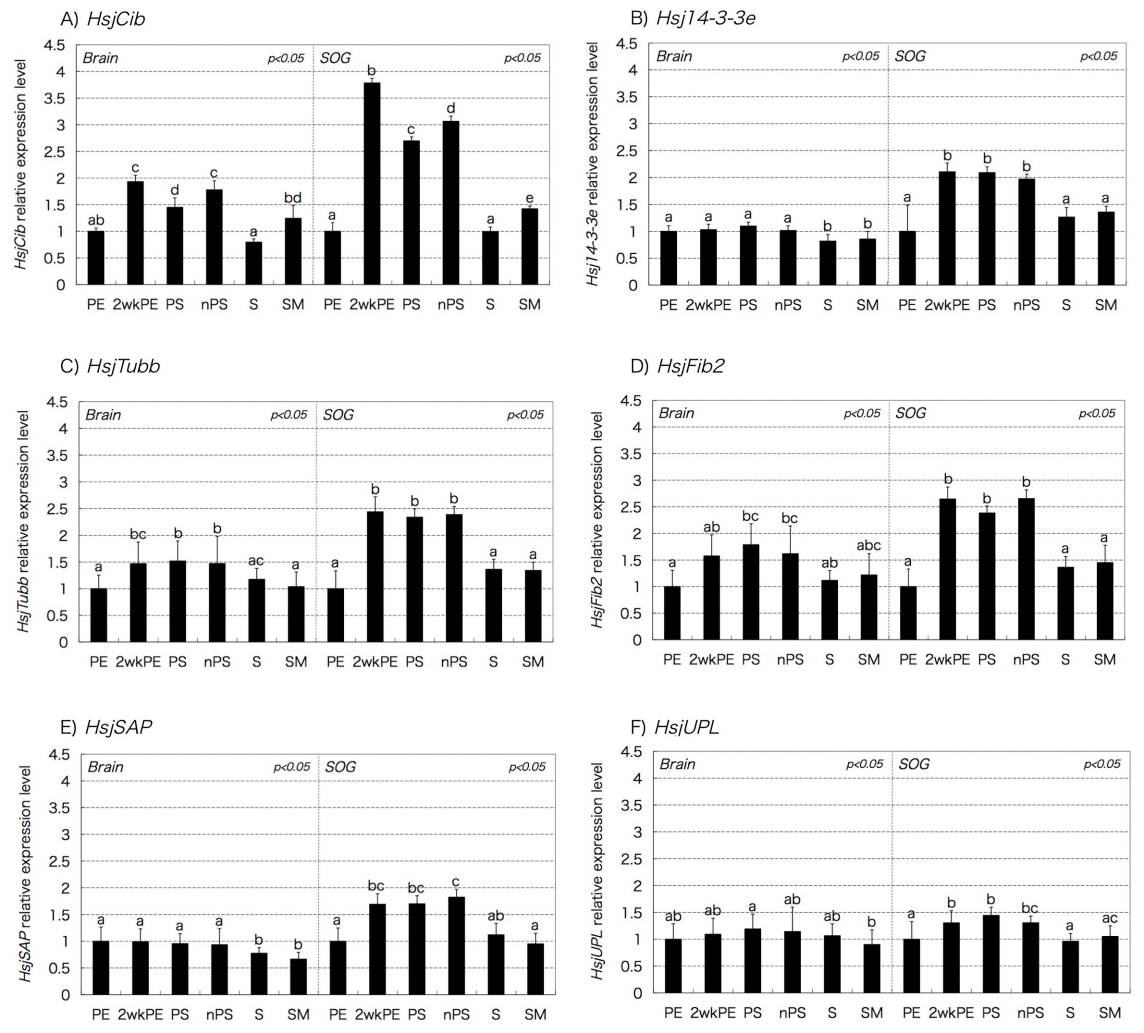
**Quantifications of candidate genes in different castes and stages**. Relative expression levels of *HsjCib, Hsj14-3-3e, HsjTubb, HsjFib2, HsjSAP*, and *HsjUPL *in different castes and stages were analyzed by qRT-PCR (second qRT-PCR). Most of the examined genes showed significantly higher levels of expressions during soldier differentiation. Y-axes indicate gene expression levels normalized based on the GAPDH expression. Experimental triplications were performed for all samples. Error bars indicate 95% confidence intervals. Statistical analysis used ANOVA with post hoc Tukey's multiple comparison tests.

The strongest differences in gene expression were observed exclusively during soldier differentiation, and not between pseudergates and differentiated soldiers (Figure 3). During the differentiation, extensive modifications to the whole body occur [24,25], and thereafter the individuals begin to show soldier-specific defensive behavior [14,16]. Considering these facts, the up-regulated genes are more likely to be associated with neuronal development than with direct behavioral differences. In addition, the gene expression differences were more distinctive in SOG than in brain, in terms of both numbers of genes and their expression levels (Figures 3 and 4). These results were consistent with our previous findings in that the SOG was much larger in soldiers than in pseudergates, and the enlargement occurred during differentiation [33].

### Identification of the up-regulated genes

Based on Gene Ontology and BLAST-X, gene names were assigned to six sequences among 13 differentiation-specific candidates (Figure 2, Additional file 1). They were provisionally named on the basis of the functions of the sequence descriptions in the BLAST results, i.e., names of putative orthologous genes, starting with the abbreviated nomenclature of the focal termite, as "*Hsj + name of sequence description*" (Additional file 1). These sequences were deposited in the GenBank/EMBL/DDBJ database under the following accession numbers: *Hsj14-3-3e*: AB511851, *HsjTubb*: AB511856, *HsjFib2*: AB511853, *HsjUPL*: AB511857. The sequences of *HsjCib *and *HsjSAP *were already described in a previous study (*HsjCib*: AB194741, *HsjSAP*: AB194732) [30]. The detailed expression patterns of these six genes were quantified again by qRT-PCR (second round of qRT-PCR) (Figure 4).

### Molecular functions of the identified genes

*HsjCib *showed the most dynamic expression profile during soldier differentiation, especially in SOG (Figure 4A). In both brain and SOG, the *HsjCib *was up-regulated before the presoldier molt, whereas at the soldier stage it returned to the same level as at the PE stage. It was reported that *Ciboulot *controls axonal growth during metamorphosis and is essential for the ellipsoid body development in *Drosophila *[34,35]. It encodes a protein with high sequence similarities to beta-thymosins, which control actin polymerization to induce structural changes of neurons [35]. In *H. sjostedti*, *HsjCib *was first reported as an up-regulated gene in developing mandibles during soldier differentiation [30]. Moreover, it was recently found that *HsjCib *was highly expressed in other tissues such as brain, muscles, and epidermis during soldier differentiation [36]. *HsjTubb *encoding beta-tubulin, one of the main components of cytoskeleton, and *HsjFib2 *encoding fibrilin 2, which constitutes microfibrils, showed similar expression patterns to *HsjCib*, although the expression profiles were not as dynamic (Figure 4C, D).

Unlike *HsjCib *and *HsjTubb*, *Hsj14-3-3e *and *HsjSAP *showed similarly unique expression patterns only in SOG during soldier differentiation (Figure 4B, E). 14-3-3epsilon is an intracellular signaling molecule belonging to the 14-3-3 protein family, which is known to be involved with neuronal migrations [37]. In terms of molecular function, 14-3-3epsilon maintains phosphorylation of NUDEL (NudE-like) and LIS1 proteins, leading to the localization change, which in turn regulates cytoplasmic dynein and kinesin functions in neurons [38,39]. In addition, it was also reported that 14-3-3epsilon controls actin polymerization in neurons by regulating MK5 (MAPK-activated protein kinase 5) [40]. Interestingly, in honeybee, 14-3-3epsilon was highly expressed in association with a genotype related to highly defensive behavior [41]. *HsjSAP *was previously reported as a gene that was highly expressed in the mandibles of *H. sjostedti *during soldier differentiation [30]. Although *HsjSAP *was named for its DNA-binding domain (known as SAP domain) that is involved in chromatin organization [42], it is difficult to speculate on its involvement in neuronal differentiation. In our study, *HsjUPL*, which was categorized as an ubiquitin-protein ligase, did not show significant expression difference during the soldier differentiation (Figure 4F).

### Neural modifications during soldier differentiation

Most of the identified genes were up-regulated in the central nervous system (CNS) during soldier differentiation, suggesting that the identified factors participate in soldier-specific neuronal modifications. Recently, it was reported that the mandibular motor neurons (MdMNs), which are located in the SOG and control the mandibles, were enlarged in soldiers in contrast to workers, and the enlargement took place before the presoldier molt [33]. In correlation with this, the up-regulated genes were more highly expressed in SOG than in brain (Figure 4). In addition, the expression patterns were consistent with the timing of the motor neuron enlargement, and some of them (*HsjCib*, *Hsj14-3-3e*, *HsjTubb*) are known to regulate cytoskeletons or motor proteins. Taken together, these genes are suggested to be involved in the motor neuron enlargement during soldier differentiation. Furthermore, these genes may indirectly affect mandibular muscle development through motoneuronal inputs, as observed in muscle formation during metamorphosis [43]. This situation is specific to termites because these developmental events associated with soldier differentiation occur through molting events, which are unlikely in social hymenopterans [7,8,16].

It is also possible that these genes may modify other neuronal networks rather than motor neurons. In honeybee workers, for example, reorganizations of mushroom bodies are known to occur in correlation with the task transitions from nursing to foraging [44]. Also in other social hymenopterans, it has been reported that histological differentiation in brain is highly correlated with task allocation or social dominance [45-47], although structural changes in interneuron connections have never been reported in termites. Thus, the up-regulated genes may be responsible for the development of unknown neuronal networks, resulting in soldier-specific defensive behavior.

## Conclusions

We compared the gene expression profiles in the nervous system between soldiers and pseudergates, and during soldier differentiation in the damp-wood termite *H. sjostedti*. The major differences in gene expression were observed during the soldier differentiation, not between the two castes. Some of the up-regulated genes are regarded as factors controlling cytoskeletal or motor proteins, suggesting that these are responsible for soldier-specific neuronal modifications. Behavioral differentiation among termite castes may occur through such neuronal modifications. This study is the first trial to analyze gene expression patterns correlated with caste-specific behaviors in termites. Further studies will reveal more detailed molecular mechanisms underlying termite polyethism and provide new insights into the evolution of social insects.

## Methods

### Termites

Colonies of *H. sjostedti *were sampled from rotten wood in evergreen forests on Yakushima Island, Kagoshima Prefecture, Japan, in May 2006 and May 2008. Colonies were kept in the laboratory as stock at approximately 25°C under constant darkness.

Termite species in the family Termopsidae lack true workers, and helper tasks are carried out by some older juveniles. These individuals are called pseudergates (etymologically: false workers) because they are socially active and potentially capable of proceeding to imagos [14,48]. Larvae undergo six molts before becoming pseudergates. Pseudergates can subsequently differentiate into either soldiers or alates, although most remain pseudergates through stationary molts under natural conditions [49,50]. Individuals that enter the alate line become nymphs first, followed by imaginal molt to alates, whereas individuals in the soldier line become soldiers through the presoldier stage [49,50].

For the screening of soldier- and/or differentiation-specific genes, termite individuals were prepared from various stages during the course of soldier differentiation, which was artificially induced by the application of juvenile hormone analogue (JHA) (Figure 1). The focused stages were defined as follows: PE (pseudergate under natural conditions), 2wkPE (pseudergate that had finished gut purge, at 2 weeks after JHA application), PS (presoldier, at 3 weeks after JHA application), nPS (presoldier under natural conditions), S (soldier under natural conditions), and SM (pseudergate before stationary molt). The individuals of PE, S, nPS, and SM were selected from the stock colony. The SM individuals were identified based on their whitish abdomens due to preparation for the next molt. There is almost no possibility that individuals undergoing soldier differentiation got mixed into this category because almost all pseudergates undergo stationary molt in normal rearing conditions in the laboratory [51].

### Soldier differentiation induced by the JHA application

The experimental procedure employed for JHA application to termites was the same as that described in Ogino et al. (1993) [22]. The JHA (pyriproxyfen) was diluted in acetone and was aliquoted into a filter paper-lined Petri dish of 60-mm diameter at a final concentration of 10 μg/dish. After evaporating the acetone, the filter paper was moistened with distilled water and 10 pseudergates were placed in each dish. Control dishes without JHA were also prepared. All of the soldier inductions were performed approximately at 25°C. Filter papers with JHA were replaced with other filter papers with JHA after 1 week. 2wkPE (the individuals that had finished gut purge before presoldier molting) and PS (the individuals that had finished presoldier molt at 3 weeks after the JHA application) were used for RNA extraction.

### RNA extraction

Fifty individuals of each stage (PE, 2wkPE, PS, nPS, S and SM) were used for the RNA extraction. Brain and SOG were isolated from all individuals, frozen in liquid nitrogen, and preserved at -80°C. Total RNA was extracted using the RNAgents Total RNA Extraction System (Promega) according to the manufacturer protocols. Colonies sampled in 2006 and 2008 were used for fluorescent differential display (FDD) and qRT-PCR, respectively.

### FDD

For the first step of gene screening (Figure 2), FDD was performed using the total RNA of the stages of PE, 2wk PE, PS, and S, essentially as described previously [26,30], with a modification of the fluorescence detection method [52,53]. Total RNA (4 μg) was treated with DNase I (Invitrogen) and then reverse transcribed using Super Script III (Invitrogen) with an anchored oligo-(dT) primer with a *Bam*H1 site (*Bam-*TG primer, 5'-CCC GGA TCC T_15 _G-3'). The conditions of reverse transcription were according to the manufacturer protocols. The resulting cDNAs were amplified by PCR in the reaction mixtures (20 μl) with 20 combinations of arbitrary 10-mers with a *Hin*dIII site (HindIII-1 to -20 primers, 5'-CGG GAA GCT TN_12_-3', where N is any base; 4 μM) [54], rhodamine-labeled Bam-TG primer (20 μM), and AmpliTaq Gold polymerase (0.5 units; Applied Biosystems). The PCR conditions were (94°C for 10 min, 37°C for 5 min, and 72°C for 5 min) for one cycle, plus (94°C for 30 sec, 55°C for 1 min, and 72°C for 1 min) for 35 cycles plus an extra 5 min at 72°C for 5 min. The PCR products were separated on a denaturing 6% polyacrylamide gel and then scanned on a fluorescence image analyzer (FluorImager; Amersham Pharmacia Biotech) and analyzed with the FluorImager595 software (Amersham Pharmacia Biotech). To ensure that the electrophoresis profile was reproducible, triplicate PCRs and electrophoresis were performed.

### Subcloning and sequencing

Bands of interest were excised, gel slices were boiled in 100 μl of distilled water, and the DNA was re-amplified by PCR with the primer combination used in FDD with an Advantage cDNA PCR Kit (Clontech Laboratories, Inc.). The PCR conditions were 94°C for 1 min plus (94°C for 30 sec and 68°C for 30 sec) for 35 cycles plus an extra 3 min at 68°C. The re-amplified DNA was subcloned into pGEM-T vector (Promega) and transfected into *Escherichia coli *JM109. The nucleotide sequences were determined with a Dye Terminator Cycle Sequencing Kit (Applied Biosystems) and an automatic sequencer (Model 3100; Applied Biosystems). Database searches for gene ontology were performed using BLAST2GO at the NCBI server (http://blast.ncbi.nlm.nih.gov/Blast.cgi).

### qRT-PCR

Because it is generally known that the FDD method tends to produce many false positives, we performed a second screening using qRT-PCR (Figures 2, 3). Among 46 sequenced candidate gene fragments, 23 cDNA fragments were selected on the basis of the fragment length because at least 100 bp is required for designing qRT-PCR primers. In addition to the focused castes/stages for the FDD analyses, the nPS and SM stages were subjected to qRT-PCR to examine whether the gene expressions were specific to soldier differentiation. Among 13 genes up-regulated during soldier differentiation, six genes identified by Gene Ontology and BLAST-X were re-quantified with qRT-PCR in technical triplicates (Figure 4).

For the second screening with qRT-PCR ("2nd screening, 1st qRT-PCR" in Figure 2), another series of RNA samples was prepared using a different colony from that used in FDD, and cDNAs were again synthesized. Total RNA from brain and SOG of all sample categories was extracted with RNAgents, and 1 μg of the total RNA was reverse transcribed using Super Script III (Invitrogen) with random hexamer primers according to the manufacturer protocols. Relative quantification of cDNAs was performed using a SYBR Green I chemistry system and ABI 7500 Fast Real-Time PCR system (Applied Biosystems). For determining endogenous control of constitutive expression, the suitability of the reference genes, i.e. *GAPDH *(glyceraldehydes-3-phosphate dehydrogenase), *18S*, and *beta-actin*, were evaluated with the appropriate software, i.e. Bestkeeper [55] and Normfinder [56]. Among those, the expression levels of *18S *and *GAPDH *were relatively stable, and the threshold cycle (Ct) of *GAPDH *was similar to those of target genes rather than *18S*; therefore, *GAPDH *gene (Accession No. AB511854) was used as the reference gene for quantification. Primers for both target and reference genes were designed using Primer Express software (Applied Biosystems; see Additional File 2). For all qRT-PCR experiments, the production of gene-specific products was assured by careful scrutiny of melting curves (conducted at the end of all qRT-PCR reactions). Data acquisition and analysis were handled by ABI Prism 7500 software v2.0.1 (Applied Biosystems). Baselines and thresholds for Ct were set automatically. For the second screening, because there were plenty of gene candidates, only one replicate of qRT-PCR per gene was performed. The genes showing expression levels greater than 1.5-fold in 2wkPE, PS, nPS, and/or S compared to PE were regarded as up-regulated genes.

After the second screening, quantifications of the up-regulated genes were again performed by qRT-PCR with technical triplicates ("2nd qRT-PCR" in Figure 2). To establish error bars, 95% confidence intervals were calculated as described in User Bulletin 2 of the ABI Prism 7700 Sequence Detection System (Applied Biosystems) using the relative standard curve method. To evaluate the significant expression differences among the stages/castes, Tukey's multiple comparison test (*p *< 0.05) was performed after ANOVA.

## Authors' contributions

YI and TM designed experiments of the study. YI, YO, AI, HM, SK carried out the molecular studies. YI performed statistical analysis. YI and TM wrote the paper. All authors read and approved the final manuscript.

## Supplementary Material

Additional file 1 **cDNA clones from differential display and match probabilities resulting from BLAST2GO search**. PDF table Click here for file

Additional file 2 **List of used oligonucleotides in qRT-PCR**. PDF table. Click here for file
