# Supplementary material for: Gene expression changes during caste-specific neuronal development in the damp-wood termite Hodotermopsis sjostedti
Source: BMC Genomics. 2010 May 20;11:314. doi: 10.1186/1471-2164-11-314 (PMC2887416; doi:10.1186/1471-2164-11-314)
Supplement: Additional file 1 — cDNA clones from differential display and match probabilities resulting from BLAST2GO search. PDF table [file 1471-2164-11-314-S1.PDF]

Sequence no.	Sequence name	Region	Stage	Size (bp)	Sequence description	Accession no.	Min. eValue	Sim mean	GO IDs	GO term
02-03, 04, 05	*HsjSAP*	SOG	S	123	heterogeneous nuclear ribonucleoprotein u-like	AB194732	1.0E-13	85.30%	GO:0003676	F: nucleic acid binding
06-19, 20, 21	*Hsj14-3-3e*	SOG	2wk, PS	219	14-3-3 protein epsilon	AB511851	1.0E-29	98.60%	GO:0001764<br>GO:0009605<br>GO:0008103<br>GO:0007444<br>GO:0021766<br>GO:0006605<br>GO:0042470<br>GO:0045172<br>GO:0007088<br>GO:0004497<br>GO:0019904<br>GO:0021987<br>GO:0046958<br>GO:0005694<br>GO:0000077<br>GO:0004863<br><br>GO:0009314<br>GO:0007093<br>GO:0019899<br>GO:0005634<br>GO:0007294<br>GO:0035308<br>GO:0005739	P: neuron migration<br>P: response to external stimulus<br>P: oocyte microtubule cytoskeleton polarization<br>P: imaginal disc development<br>P: hippocampus development<br>P: protein targeting<br>C: melanosome<br>C: germline ring canal<br>P: regulation of mitosis<br>F: monooxygenase activity<br>F: protein domain specific binding<br>P: cerebral cortex development<br>P: nonassociative learning<br>C: chromosome<br>P: DNA damage checkpoint<br>F: diacylglycerol-activated phospholipid-dependent protein kinase C inhibitor activity<br>P: response to radiation<br>P: mitotic cell cycle checkpoint<br>F: enzyme binding<br>C: nucleus<br>P: germarium-derived oocyte fate determination<br>P: negative regulation of protein amino acid dephosphorylation<br>C: mitochondrion
09-49, 51	*HsjFib2*	B, SOG	2wk, PS	186	fibrillin 2	AB511853	1.0E-18	62.20%	GO:0008150<br>GO:0005576<br>GO:0005201<br>GO:0005488<br>GO:0005509<br>GO:0035108<br>GO:0005578<br>GO:0030326	P: biological process<br>C: extracellular region<br>F: extracellular matrix structural constituent<br>F: binding<br>F: calcium ion binding<br>P: limb morphogenesis<br>C: proteinaceous extracellular matrix<br>P: embryonic limb morphogenesis
17-07	*HsjTubb*	SOG	2wk, PS	144	beta-tubulin	AB511856	1.0E-21	99.85%		
25-20	*HsjUPL*	SOG	2wk	125	ubiquitin-protein ligase	AB511857	1.0E-15	87.60%		
25-21, 22, 24	*HsjCib*	SOG	2wk	126	HsjCiboulot	AB194732	1.0E-17	77.20%	GO:0003779<br>GO:0005737<br>GO:0007010	F: actin binding<br>C: cytoplasm<br>P: cytoskeleton organization and biogenesis
