# Supplementary material for: Gene expression changes during caste-specific neuronal development in the damp-wood termite Hodotermopsis sjostedti
Source: BMC Genomics. 2010 May 20;11:314. doi: 10.1186/1471-2164-11-314 (PMC2887416; doi:10.1186/1471-2164-11-314)
Supplement: Additional file 2 — List of used oligonucleotides in qRT-PCR. PDF table. [file 1471-2164-11-314-S2.PDF]

List of used oligonucleotides in qRT-PCR

Seq. no.	Seq. name	Primer (5' - 3')	
02-02		GCGCACAAGACAGGAGTACCT	AAAATTTCTGGCCTGGATCAAC
02-03, 04, 05	*HsjSAP*	TGGAGCTTCGTGCCGAAT	CTGTCAATCAGAGTGGCTTTGACT
06-18		ACTGGCAATTGTAAGACAGAGGAA	CCCCTACGTATAAGAAGTTTTGTTAACA
06-19, 20, 21	*Hsj14-3-3e*	TGGCGGAGCAAGCTGAA	CATCCAGTGACGCTACCTTCTTC
09-49, 51	*HsjFib2*	CCGTGTCGAGGCAATGC	AACCTTTTGGACACTGACACTCAA
09-52		TCCAGCATGCAACAGAATGTC	GAGTACCACCTAAATCTGTGTTGGAA
09-53		CACCGACTAGACGTCATAGCTCTTC	CCACCGGTCAGTCTAATATGAAAA
12-65,66,67,68		AACGTAGACGGTTAGCTCGAGAA	CTCTGTTCCTCTTCCAATCTTTGTC
13-01		CCCTTGGGTGTGGATGTCA	TCCGCAAAATCATGCTTCTG
13-03		GACGCAACAAGGCAGACAAAT	GCCATGTCAAGCGGTGAGTA
13-04,05		TCACAGCTCCTGTATGTGGTACAA	TGAGAATTCAAAATGGAGGTATAGGTT
16-17,19,20,21		CATTGTACGACGATTTCCTAGCAT	TGTTTTGGTTCGCTTCACATG
17-07	*HsjTubb*	GAACCCGGGACCATGGAT	CGTAGTTGTCGGGTCTGAACAG
17-08,10,12		ACAGAGAGAATACAAATATCTCCAAAGAAG	TTTCCAATTGACATTTCCTCTTTCA
17-09		CATTGTACGACGATTTCCTAGCAT	TGTTTTGGTTCGCTTCACATG
23-33		CACATGCTGTGAGCTCGATGA	AGGATGGTCCCCCCATATTC
23-34		GGAGAGGCTAATGGAGATGATCA	ACCTCCCCTTTTTAGCACATTCT
25-19		TCAAACACCACCACCATCTAAGG	CCCAAATGACCAATTATCATATGGTT
25-20	*HsjUPL*	ACTCCACTTTGTGTCTGGGTTGT	CCGCTAGAGGCAAAGCTTCA
25-21, 22, 24	*HsjCib*	ATCCGGCCATTAGACAACGA	TTGGATAAACCACCATGGCA
27-25		TTATGCTTCACTTCGTATCCTACGA	TCTCCGCCATCTTTGGATCT
27-27		GAACCCATGTGGTTTCTGCAA	AGGACATGTTGAGAAACCAGCTTA
27-28		CATGGTGCTGCAGTGTGTTG	TTTCTTCGCAGCTGTAATGTCACTA
	HsjGAPDH	TGAGTCATACAACCCATCATTGAAG	TTAGCAAGAGGTGCCAAGCA
